# Overcommercialisation of dental practice in England and decline in oral Health

**DOI:** 10.1136/bmj-2025-088509

**Published:** 2026-04-28

**Authors:** Rizwana Lala, Lisa M Jamieson, Sonia Saxena

**Affiliations:** 1 The University of Sheffield, Sheffield Centre for Health & Related Research (SCHARR) School of Medicine and Population Health; 2 The University of Adeliade, Australian Research Centre for Population Oral Health, Adelaide Dental School; 3 Imperial College London, School of Public Health

In England, there is simply *no universal coverage*, and *no right to dental healthcare*. Currently 97% of people without an NHS dentist cannot access one, and over 30,000 children a year have teeth extracted under general anaesthetic due to the lack of preventative dental care.^1,2^

In many countries, dentistry evolved separately to medicine and England is no exception. Most dental practices operate as for-profit commercial entities, ranging from small businesses to corporates, including within universal healthcare systems like the UK’s National Health Service (NHS). Using the commercial determinants of health lens our analyses focus on how the place of dentistry within the NHS evolved to drive over-commercialisation and entrench long-standing oral health inequities.

## How the place of dentistry evolved within the NHS

In 1948, after the ravages of war, the NHS was built with an almost utopian socialist vision — public, universal, and free at the point of need. A vision that was short-lived and never fully realised. Although free at the point of need for everyone, dental practices remained private businesses holding contracts with the NHS. Demand for dentistry exceeded government budget estimates. So just three years after the inception of the NHS, the chancellor introduced patient charges for dentistry. This policy was bitterly opposed by Aneurin Bevan, the chief architect of the NHS, and prompted his resignation from the Cabinet. Besides the exception of specific groups such as children, pregnant women, and some individuals receiving benefits, NHS dentistry is no longer free and levies patient charges for *all* services necessary to maintain oral health, from routine to urgent care. NHS dentistry does not include cosmetic dentistry.

This separation from mainstream health services, private contractor model and patient charges has led to a matured infrastructure and culture for over-commercialisation in dentistry. Unlike the wider NHS, dentistry operates within a ‘mixed economy’, whereby NHS and private dentistry operate side-by-side in for-profit dental practices. The same patient can receive both NHS and private treatments. For example, a patient may opt for an NHS dental check-up and private cosmetic teeth whitening and pay the relevant — NHS and private — invoices. Thus, in dentistry, social norms have shifted, whereby individuals pay; something that would be unacceptable for wider healthcare.

Since the 1990s, corporate dental chains have expanded in UK dentistry through acquisitions and mergers, securing a growing share of NHS dental contracts. Dental practices could establish anywhere, with NHS income linked to patient numbers and procedures performed.

In 2006, the government introduced a budget cap on NHS dentistry. If patient demand exceeds NHS dental capacity, the NHS is not obliged to fund additional services. Consequently, patients, including children lost their political right to access NHS dentistry.^3^ NHS dental services cover half the population, and this coverage is declining. In contrast, dental practices can offer private care without limits, treating as many patients as they choose. Thus, patients report long waits (sometimes years) for NHS dentistry, but early appointments for private care at the same practice.^4^

Austerity policies, precipitated by the 2008 financial crash, resulted in cuts to the NHS as a whole. But cuts to NHS dentistry are the deepest. In England, the NHS dental budget has remained static at *£*3bn over the past 15 years— a real-term plummet of 26%.^5^ With successive funding shortfalls, some NHS dental treatments are provided at a loss. Inflation-busting increases in patient charges^5^ and cross-subsidy from higher-profit private treatments keeps NHS dentistry afloat.^6^ This model favours dental practices in affluent areas and epitomises the ‘Inverse Care Law’, whereby there are ‘dental deserts’ in poorer areas with greater needs.^7^

With an ever-increasing funding squeeze, some practices are giving up their NHS dental contracts, thus returning the funding to deliver NHS care and focussing solely on private dentistry. This means patients at these practices can no longer access NHS care and must either find another NHS dentist or pay for private care. A commercial sector even exists to offer guidance on transitioning NHS dental practices to fully private.

Dental chains have the power to make strategic corporate decisions that have huge impacts on dental access. For example, in 2023, one corporate gave up 85 NHS dental contracts abandoning thousands of patients without NHS healthcare.^8^ Over the past two years, dental practices have returned *£*900 million of NHS dental funding.^9^ So despite high demand for NHS dentistry, in some areas, a third of the stagnant dental budget is underspent.^10^ NHS dental provision is at an all-time low. Almost everyone (97%) without an NHS dentist already, cannot access one.^2^

High NHS charges are another major barrier to accessing care, hitting the poorest and ethnic minority communities the hardest.^4^ In 2026, in England, NHS dental charges range from *£*27.90 for a check-up to *£*332.10 for a denture. Moreover, limited NHS dental coverage may have enabled practices to hike their private charges, rising to 22% in just two years, prompting an investigation by the Competition and Markets Authority.^9^ Patients consistently report delaying or foregoing dental treatments due to costs.^11^

## Consequences for Oral Health Equity

The disconnect between dentistry and wider healthcare, combined with its delivery through private businesses has created gaps in patients’ awareness of their NHS entitlements.^12^ Dental practices have more power to cherry pick who to treat and what treatments to offer. Patients depend on dentists for information about what NHS dental treatments are available within a contract that under-remunerates prevention, complex care, and caps annual payments. This information asymmetry between patients and dentists, the need for cross-subsidy, and the relative power of dental practices as commercial businesses render dentistry vulnerable to market failures with implications for equitable access and quality of care. A 2026 study demonstrates dental practices are less likely to offer complex treatments like molar endodontics on the NHS to ethnic minority communities, instead offering simpler options like tooth extractions.^13^ Dentists may induce demand for private treatments, which are more profitable than NHS dentistry such as cosmetic teeth whitening, aligners, and even Botox and filler injections.

Insufficient NHS dentistry compounded by patient charges reduces opportunities to prevent oral diseases and increases the likelihood of patients needing complex care. Preventable oral health inequities are evident in children as young as three through to adults. Those from deprived backgrounds disproportionately suffer from periodontal (gum) diseases. Incidence rates and deaths from oral cancer are rising along classed and racial lines. Dental caries (tooth decay) is socio-economically and racially patterned. Also, demand can be displaced onto wider NHS services which are free at the point of need, including GPs and A&E. This risks poorer care, inappropriate antibiotic prescriptions, antimicrobial resistance and cost-ineffective pressures on the wider health service (Table 1).

Behind this crisis lie human stories that impact not just oral health, but overall health and wellbeing. Trauma of childhood dental extractions, disruptions to school and family life, pain, swelling, stigma, and even life-threatening dental infections like septicaemia and Ludwig’s angina.^18^ There is another dimension to the over-commercialisation of dentistry deserving attention.

## Expanding Commercialisation of Dentistry

**Figure 1:**
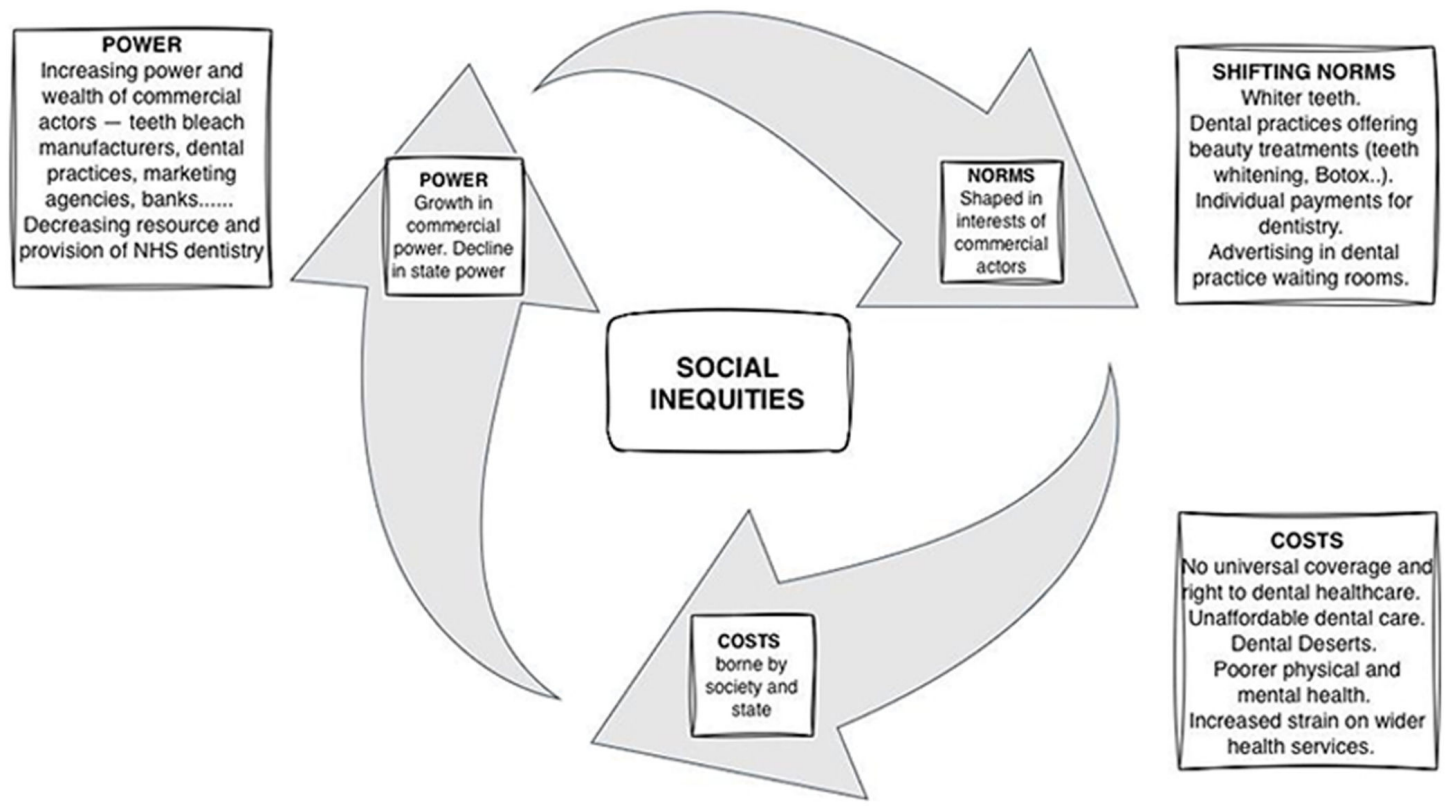
Commercial actors increasing their power and deepening the cycle of oral health inequities Adapted from Gilmore et al. (2023) Lancet Commercial Determinants of Health Series^17^

Beyond dental practices, financial institutions, private equity firms, insurers, marketing agencies all play a role in shifting cultural norms to increase their market power (Figure 1). Lower dental need in affluent areas incentivises practices to upsell cosmetic dentistry. Marketing and consumer culture shapes perceived treatment needs. Until 1997, dental practices were banned from promotional marketing, but these restrictions have gradually relaxed. Consultancy and marketing firms advise dental practices how to increase profit margins through marketing and uptake of higher-profit cosmetic treatments and improve financial performance.

Dentistry’s commercialisation extends beyond individual upselling, encompassing wider political and economic relationships, interacting with multi-national commercial actors. Between 1992 and 2001, a multi-national manufacturer of teeth bleach, together with its distributor sought to introduce higher-strength (>0.1% peroxide) teeth whitening products into the UK and European markets.^20^ Ultimately, the British Dental Association, dentists’ trade union, representing dentists, and therefore, dentist-owned dental practices, engaged with the European Commission through the European Council of Dentists. This led to the removal of legal restrictions on higher-strength teeth whitening products, thereby opening European markets to cosmetic teeth whitening undertaken in dental practices.^21^

Dental practices often promote elective teeth whitening and cosmetic dentistry in waiting rooms and across online platforms like Instagram. This has had huge ramifications, increasing social pressures to conform to a singular straight, white smile. Such pressures disproportionately impact women and ethnic minority communities due to the dominance of patriarchal norms and white-centric beauty standards.^21^

The UK cosmetic dentistry market has outgrown the entire NHS dentistry budget and is relentlessly rising.^22^ This encompasses treatments that lie out of scope for dentists such as skincare. One in four practitioners administering cosmetic injectables are dentists.^23^ Shifting social norms are making people unhappier with their dental and facial appearance. In 2006, over half of the UK population was concerned with their dental appearance. Now, in less than a decade, more than three quarters are self-conscious.^24^ Nearly a third of under 35-year-olds have had cosmetic dentistry, some spending over *£*25,000.^25^ Therefore, dental practices offer finance plans and patients are incurring debt. Financialisation, patterns of profit generation through financial institutions is a feature of contemporary over-commercialisation. In dentistry, financialisation includes growth of private equity such as corporate acquisitions of practices, services operating for profit e.g. denial of unprofitable complex treatments, and inducement of debt-driven consumer markets such as elective cosmetic care.

There is greater social acceptance of private dentistry, individual payments for healthcare, promotional marketing and upselling unessential cosmetic treatments within healthcare spaces (NHS and private dental practices). The information asymmetry between dentists and patients can result in people making underinformed decisions about their health. Physical harms of cosmetic dentistry, e.g. veneers and crowns include soft tissue injuries, irreversible tooth damage, and even tooth loss. Idealised photographs of body images, and financial debt are factors influencing mental health.^26^ Remedial care from physical and mental harms can place additional pressures on broader NHS services. So the over-commercialisation of dentistry contributes to inequities in dental access, poorer physical and mental health across the social spectrum and increased strain on the wider health service.

By shaping social norms to grow profits and power, commercial actors, including dental practices deepen historically entrenched inequities. These processes exemplify the commercial determinants of health (Figure 1).

## How can the dental system promote health and equity?

Although the NHS faces significant financial pressures, the UK Government’s 10-Year-Plan for Health recognises insufficient dental access is driving poor oral health and increasing pressure on hospitals. Therefore, widening access to NHS dentistry and improving the NHS dental contract are core policy commitments^27^ and may provide cost-effective solutions to improving oral health. This reflects broader consensus across many countries that access to dentistry is a political right. Brazil pursued an ambitious integration of dentistry into primary care offering universal free access^18^ which was strengthened as legal right in 2023. Scotland introduced universal free dental examinations and abolished patient charges for under 26-year-olds. In 2023-24, Scotland’s NHS dentistry budget was *£*476 million — 3.9% of the total health budget (*£*12.1 billion), compared with 3% in England reflecting higher overall health spends per capita and a larger proportion allocated to dentistry. Patients also have the right to registration, with 96.3% of the Scottish population registered with an NHS dentist.^28^ The Scottish government has pledged universal free dental access by the end of this parliamentary term, which based on current receipts will cost an additional *£*75 million.^29^

Underpinned by legal frameworks, dentistry must be integrated with wider healthcare to improve oral health outcomes and equity. Global studies and decade-long NHS dental contract pilots provide evidence-based recommendations for improving oral health, emphasising prevention, quality of care and equity.^18,30^ Table 2 lists the factors that drive the over-commercialisation of dentistry and recommendations for change.

## Wider Lessons for the Healthcare System

The over-commercialisation of dentistry has had detrimental consequences for equitable dental access and health outcomes. Patient charges have been muted for NHS GP and hospital appointments. The poly-crisis of dentistry — no universal coverage, unaffordability, physical and mental health inequities across social groups — exacerbated by a growing private sector should serve as a prescient warning against deregulating and commercialising broader NHS services.

## Figures and Tables

**Table 1:** Oral Health Inequities in England and Prevention Opportunities with Primary Care Dentistry ^14–19^

Oral Health Inequity	Prevention and Opportunities to Improve Health Outcomes with Primary Care Dentistry
**Dental Caries (Tooth Decay)** Children living in deprived areas and some ethnic minority children have significantly higher levels of dental caries in primary (baby) and permanent (adult) teeth^1,15^ Majority of dental decay in children is untreated with fillings^15^ Decayed teeth is the most common reason for childhood hospital operations and general anaesthetics (over 30,000 every year)^1^	Regular fluoride varnish applications in children and adolescents can prevent dental caries by up to 37% in baby teeth and 43% in adult teeth Prevention and early treatment with fillings can reduce severe pain, sleepless nights, missed school days, disruption to family life, appearance-based bullying, stigma, and body image-related mental health ramifications^17,18^ Prevention and early treatment can avoid pressures on the wider health service by reducing hospital extractions, inappropriate antibiotic prescriptions and A&E admissions due to life-threatening dental infections (e.g. Ludwig’s angina and septicaemia)^14,18^
**Periodontal Diseases** ^19^ Majority of adults have periodontal (gum) conditions 12% suffer severe periodontal diseases requiring complex treatment More common in men, older people and those from deprived backgrounds	Periodontal screening and early treatment can prevent the need for complex treatment and tooth loss
**Oral Cancer**^15^ Incidence and mortality rates Increasing Doubles across deprivation quintiles Variations amongst ethnic minority communities 52% cases present late (stage IV)	Opportunistic oral cancer screening during routine dental examinations can reduce morbidity (difficulty eating, drinking, facial disfigurement, metastasis) and mortality (death). Early diagnosis improves oral cancer 5-year survival from 50% up to 80%

**Table 2:** Factors driving the over-commercialisation of dentistry and recommendations for change ^18,30^

Factors driving over-commercialisation	Recommendations for change
*Separation of dentistry from wider healthcare.* *Private healthcare* (dentistry) operating alongside NHS dentistry at the same practice — mixed economy	*Universal right to healthcare* (including dentistry), free at the point of need. *Integrate dentistry with wider primary care services and provide parity of access to care.* Restore right to registration and continuity of care for children and adults
*Dental remuneration focused on delivery of dental procedures*	*Embed prevention to focus on oral and overall health outcomes* Remuneration for prevention. Use of full dental workforce, including extended duty dental nurses to deliver cost-effective oral and general health prevention e.g. fluoride varnish applications, and signposting to wider prevention such as tobacco cessation, alcohol reduction, mental health services
*Fiscal austerity —* budget cuts, underfunding of NHS dentistry	Legislative protections for oral health funding *State funding* for healthcare is legally protected from economic fluctuations Funding for dentistry to keep pace with inflation Remove reliance on patient charges and cross-subsidy from private care to fund NHS dentistry
*High pressure on the workforce —* NHS contract remuneration based largely on activity (dental procedures (‘treadmill’ dentistry) Under-remuneration for prevention and complex treatments	*Strengthen labour rights* Fair remuneration for complex treatments such as endodontics and periodontal treatments Co-produced NHS dental workforce plan to promote staff recruitment and retention, and thereby improving quality and continuity of care
*Information asymmetry* between patients and dentists Greater flexibility for dental practices to decide who to treat and what procedures to undertake	Clear standardised patient information and legal protections of patient entitlements to NHS dental care Training for dental teams in shared decision making and informed consent, including effective communication about health benefits, short- and long-term health risks, and short- and long-term financial costs.
*Personal responsibility* — patient charges and responsibility to seek care (NHS or private)	*Public health approach to reduce health inequities* High availability of prevention-focused services particularly in under-resourced, high deprivation areas through weighted capitation based on deprivation and need Actively encourage dental attendance in children and vulnerable adults
*Market freedoms* Removal of advertising restrictions Promotions and provision of services that lie outside the scope of practice of dentistry (Botox, fillers, skincare)	*Rebalance market freedoms* Advertising bans, especially of before and after cosmetic dentistry images that cause body confidence issues Regulate dentists’ cosmetic practice that lies outside their General Dental Council recognised scope of practice e.g. Botox and filler injections and skincare
*Financialisation —* finance plans with the dentist	*Price controls* for private dentistry

## Key Messages

- There is no right to access NHS dental healthcare in England, even for children
- NHS dentistry has long been funded through a combination of state support and patient charges. Successive state underfunding of NHS dentistry, along with a weakly regulated private sector is fuelling rising unaffordability and inequitable dental access, especially for deprived and minority communities
- An insufficiently regulated private sector is driving the uptake of costly cosmetic dentistry which can heighten body image concerns, financial strain and mental health problems
- Limited access to affordable dentistry is causing people to delay treatments and shift demand onto wider healthcare services that are free at the point of need such as GPs and A&E, resulting in poorer care, and costly pressures on the health system
- England should treat dental healthcare as a universal right, giving it parity with wider primary care by integrating services, abolishing patient charges, and properly funding preventive care, following models in Scotland and Brazil
